# Membrane-displayed somatostatin activates somatostatin receptor subtype-2 heterologously produced in *Saccharomyces cerevisiae*

**DOI:** 10.1186/2191-0855-2-63

**Published:** 2012-11-30

**Authors:** Keisuke Hara, Tomohiro Shigemori, Kouichi Kuroda, Mitsuyoshi Ueda

**Affiliations:** 1 Division of Applied Life Sciences, Graduate School of Agriculture, Kyoto University, Sakyo-ku, Kyoto 606-8502, Japan

**Keywords:** Membrane-displayed ligand, PepDisplay, Yeast GPCR assay, Cyclic peptide, Somatostatin receptor subtype-2, Chimeric Gα protein

## Abstract

The G-protein-coupled receptor (GPCR) superfamily, which includes somatostatin receptors (SSTRs), is one of the most important drug targets in the pharmaceutical industry. The yeast *Saccharomyces cerevisiae* is an attractive host for the ligand screening of human GPCRs. Here, we demonstrate the utility of the technology that was developed for displaying peptide ligands on yeast plasma membrane, termed “PepDisplay”, which triggers signal transduction upon GPCR activation. A yeast strain that heterologously produced human somatostatin receptor subtype-2 (SSTR2) and chimeric Gα protein was constructed along with membrane-displayed somatostatin; somatostatin was displayed on the yeast plasma membrane by linking it to the anchoring domain of the glycosylphosphatidylinositol anchored plasma membrane protein Yps1p. We demonstrate that the somatostatin displayed on the plasma membrane successfully activated human SSTR2 in *S. cerevisiae*. The methodology presented here provides a new platform for identifying novel peptide ligands for both liganded and orphan mammalian GPCRs.

## Introduction

G-protein-coupled receptors (GPCRs) are proteins that possess seven transmembrane domains and constitute one of the largest gene families in the human genome (Jacoby et al. 2006). More than 50% of all drugs, with annual worldwide sales exceeding $50 billion, regulate GPCR functions, and approximately 30% of these drugs directly target GPCRs (Hopkins and Groom 2002; Xiao et al. 2008). Although the human genome contains 720–800 GPCRs (Siehler 2008), it is still less than 50 GPCRs that are the targets of commercially available drugs (Eglen et al. 2007). The development of a robust, reliable, and cost-effective functional screening method for both known and orphan GPCRs is a major focus of the pharmaceutical industry (Thomsen et al. 2005). Accordingly, it is evident that the market for the discovery and development of drugs targeting GPCRs will continue to grow in the coming decade.

A GPCR assay that uses the baker’s yeast *Saccharomyces cerevisiae* has been established as an experimental system for characterizing human receptor pharmacology and signal transduction mechanisms (Dowell and Brown 2002). Its attractive features are its simplicity with respect to genetic manipulation, economical propagation, easy maintenance of stably expressing cell lines, availability of an evolutionarily highly conserved signaling pathway, and presence of only two endogenous GPCRs that can be readily eliminated (Ladds et al. 2005). In spite of the major differences in G-proteins and membrane lipid composition between yeasts and mammalian cells, numerous cases of successful production of functional mammalian GPCRs in yeast have been documented (Xue et al. 2008). In addition, yeasts are attractive hosts for the high throughput screening of peptides or proteins from large combinatorial libraries (Chao et al. 2006; Li et al. 2007; Ueda 2004), with a high transformation efficiency of plasmids of up to 1–1.5 **×** 10^8^ transformants/μg achieved (Benatuil et al. 2010). Furthermore, homologous recombination (Ito et al. 2008), also known as gap-repair cloning, can be employed in yeast, which supports the reliable and cost-effective construction of random plasmid libraries.

The membrane-tethered peptide ligand is rather a new concept that was first documented by Fortin et al. (2009). In yeast, the display of heterologous peptides or proteins on cell wall was established by fusion with a part of cell wall proteins (Ueda and Tanaka 2000). However, it is considered that the displayed peptide ligand needs to localize within the range allowing the access to membrane-localized GPCRs. Therefore, we displayed the peptide ligand on the plasma membrane rather than cell wall, by using an anchoring domain of the glycosylphosphatidylinositol (GPI)-anchored plasma membrane protein, Yps1p. In the previous study, the α-factor, which is a 13-amino acid residue-long peptide agonist of the yeast pheromone response pathway, was successfully displayed on the yeast plasma membrane, and signal activation was observed by employing a fluorescent reporter gene assay (Hara et al. 2012a). Extended application of this system to human GPCRs, which comprise one of the most important types of drug targets that are being pursued today, would be a valuable technology.

Somatostatin is a naturally occurring gastrointestinal hormone that regulates various endocrine and exocrine processes (Patel 1999). It is a cyclic tetradecapeptide with a disulfide bond between Cys^3^ and Cys^14^ residues. Cyclic peptides are promising scaffolds for peptide therapeutics because cyclic structures increase not only the physiological (serum) stability of peptides but also their affinity for binding partners owing to their conformational rigidity (Katoh et al. 2011). In fact, somatostatin receptor ligands, most of which possess cyclic structures, have been widely used as safe and effective treatments for pituitary and neuroendocrine tumors (Low 2004; Patel 1999; Weckbecker et al. 2003). The effects of somatostatin are transduced by its binding to plasma membrane-localized somatostatin receptors (SSTRs). SSTRs are encoded by five subtypes (SSTRx; x = 1–5) and compose a subset of the GPCR superfamily (Jacoby et al. 2006).

In this study, the range of application of our technology for displaying peptide ligands on yeast plasma membranes, namely “PepDisplay”, was extended to the activation of human GPCR that was heterologously produced in *S. cerevisiae*. The methodology presented in this study could be useful for identifying novel peptide ligands for both liganded and orphan mammalian GPCRs.

## Materials and methods

### Strains and media

The haploid yeast (*S. cerevisiae*) strain W303-1A (*MAT*a, *ade2-1*, *his3-11,15*, *leu2-3,112*, *trp1-1*, *ura3-1*) (Thomas and Rothstein 1989), and other strains that were constructed specifically for our study, and their genotypes are listed in Table 1. The yeast strains were cultivated aerobically in YPD medium (1% [w/v] yeast extract, 2% [w/v] peptone, and 2% [w/v] glucose), or in synthetic complete (SC) medium lacking histidine, adenine, leucine, and tryptophan (SC-HALW), containing 0.67% (w/v) yeast nitrogen base without amino acids, 2% glucose, Multiple Drop-outs (DSCK292; ForMedium, Norfolk, UK), and appropriate amino acids. The assay medium was buffered at pH 7.2 with 50 mM PIPES. Agar (2% [w/v]) was added to produce YPD and SC solid media. *Escherichia coli* DH5α (Toyobo, Osaka, Japan) was used as the host for recombinant DNA manipulation and grown in Luria–Bertani medium (1% [w/v] tryptone, 0.5% [w/v] yeast extract, and 0.5% [w/v] sodium chloride) containing 100 μg·mL^-1^ ampicillin. After selection, a single colony was used to isolate the plasmid for transforming the yeast.

**Table 1:** List of yeast strains used and constructed in this study

**Strain**	**Genotype**	**Reference**
W303-1A	*MAT*a; *ade2-1; his3-11,15; leu2-3,112; trp1-1; ura3-1*	(Thomas and Rothstein 1989)
WH-3	*MAT*a; *ade2-1; his3-11,15; leu2-3,112; trp1-1; ura3-1; sst2*ΔΔ; *far1*ΔΔ	(Hara et al. 2012a)
WH-7	*MAT*a; *ade2-1; his3-11,15; leu2-3,112; trp1-1; ura3-1; sst2*ΔΔ; *far1*ΔΔ; *ste2*ΔΔ	Present study
WH-9	*MAT*a; *ade2-1; his3-11,15; leu2-3,112; trp1-1; ura3-1; sst2*ΔΔ; *far1*ΔΔ; *ste2*ΔΔ; *gpa1*ΔΔ; p413-GPA1(Gi2)	Present study
WSTH-9	*MAT*a; *ade2-1; his3-11,15; leu2-3,112; trp1-1; ura3-1; sst2*ΔΔ; *far1*ΔΔ; *ste2*ΔΔ; *gpa1*ΔΔ; p413-GPA1(Gi2), p412-SSTR2, pYEXfet	Present study

### Expression vectors

PCR was performed using KOD-Plus-DNA polymerase (Toyobo).

Multistep construction of the SSTR2 expression plasmid, p412-SSTR2, was initiated by inserting an *Sac*I-*Kpn*I fragment of the *GPD* promoter and *CYC1* terminator from p413-GPD (Mumberg et al. 1995) into *Sac*I and *Kpn*I sites in pRS412 (Brachmann et al. 1998) to create p412-GPD. The DNA fragment encoding human SSTR2 was amplified from a human brain cDNA library (B1234035; BioChain, Hayward, CA, USA) with primers 5^′^-CGCggatccAAAAAAATGGACATGGCGGATGAGC-3^′^ and 5^′^-TGCGgtcgacTCAGATACTGGTTTGGAGGTCTC-3^′^. This fragment was inserted into the *Bam*HI and *Sal*I sites of p412-GPD to create p412-SSTR2.

The chimeric Gα-producing plasmid, p413-GPA1(Gi2), was constructed as follows. The DNA fragment that consists of the *GPA1* promoter and the *GPA1* ORF was amplified from W303-1A genomic DNA using primers 5^′^-CGCgagctcATGTGCATTAAAGCAGTAATGATAAGACG-3^′^ and 5^′^-GCGctcgagTCAAAACAAACCACAATCTTTAAGGTTTTGCTGGATGATTAGATCGG-3^′^. This chimeric Gα-encoding sequence was inserted into the *Sac*I and *Xho*I sites of p413-CYC (Mumberg et al. 1995), from which the *Sac*I-*Xho*I fragment of the *CYC* promoter was removed, to create p413-GPA1(Gi2).

Plasmids for producing membrane-displayed somatostatin with different linker lengths (Table 2) were constructed as follows. DNA fragments with different linker lengths were amplified from pYS0 (Hara et al. 2012a) using the following primers: 5^′^-GCGggatccGGTGGATCTGATTACAAGGATGACGATGACAAG-3^′^, where the underlined linker sequence varied depending on the linker length (namely, (GGTGGATCT)_n_GGT (n = 0, 1, 2, 3, 4, 7, 13) for linker length 1, 4, 7, 10, 13, 22, 40; (GGTGGATCT)_n_GGTGGA (n = 0, 1, 2, 3, 6) for linker length 2, 5, 8, 11, 20; (GGTGGATCT)_n_ (n = 1, 2, 3, 4, 5, 7, 10) for linker length 3, 6, 9, 12, 15, 21, 30), and 5^′^-TGGCCGAgtcgacTCAGATGAATGCAAAAAGAAGAGAAATTAATG-3^′^. The fragments were then inserted into the *Bam*HI and *Sal*I sites of pYS0, resulting in plasmids pYS1, pYS2, pYS3, pYS4, pYS5, pYS6, pYS7, pYS8, pYS9, pYS10, pYS11, pYS12, pYS13, pYS15, pYS20, pYS21, pYS22, pYS30, and pYS40. Next, a DNA fragment encoding the glucoamylase secretion signal and somatostatin was amplified from pULD1 (Kuroda et al. 2009) with primers 5^′^-CGGggtaccATGCAACTGTTCAATTTGCCATTG-3^′^ and 5^′^-CGCggatccCCACAGGATGTGAAAGTCTTCCAGAAGAAATTCTTGCAGCCAGCGGCAGAAACGAGCAAAGAAAAG-3^′^. It was inserted into the *Kpn*I and *Bam*HI sites of the plasmids above. The resulting plasmids were designated pYS0-sst, pYS1-sst, pYS2-sst, pYS3-sst, pYS4-sst, pYS5-sst, pYS6-sst, pYS7-sst, pYS8-sst, pYS9-sst, pYS10-sst, pYS11-sst, pYS12-sst, pYS13-sst, pYS15-sst, pYS20-sst, pYS21-sst, pYS22-sst, pYS30-sst, and pYS40-sst, respectively. Additionally, the DNA fragment encoding the glucoamylase secretion signal sequence, but lacking the somatostatin sequence, was amplified similarly with primers 5^′^-CGGggtaccATGCAACTGTTCAATTTGCCATTG-3^′^ and 5^′^-CGCggatccACCGGCAGAAACGAGCAAAGAAAAGTAAG-3^′^. It was then inserted into the *Kpn*I and *Bam*HI sites in pYS0. The resulting plasmid was named pYS0-nega and was used as a negative control (Table 2).

**Table 2:** Constructed plasmids for membrane display of somatostatin in this study

**Plasmid**	**Description**
pYS_n_-sst (n = 0, 1, 2, 3, 4, 5, 6, 7, 8, 9, 10, 11, 12, 13, 15, 20, 21, 22, 30, 40)	Membrane display of somatostatin with FLAG tag and various linker lengths
pYS0-nega	Membrane display of FLAG tag (same construct as pYS0-sst but lack somatostatin sequence, and used as a negative control)
pYS-notag3-sst, pYS-notag20-sst, pYS-notag38-sst	Membrane display of somatostatin without FLAG tag
pYS21-sst(W8A), pYS21-sst(C14A)	Membrane display of alanine-substituted somatostatin with FLAG tag

Plasmids for producing membrane-displayed somatostatin without FLAG tag (Table 2) were constructed as follows. DNA fragments that encoded the linker region and the anchoring domain of Yps1p were amplified from W303-1A genomic DNA with primers either 5^′^-GCGggatccACATCAAGTAAAAGAAATGTTGGTGATC-3^′^, 5^′^-GCGggatccGGCGGTAGTGGAGGCAGCGGAGGCTCGGGAGGTTCAGGAGGCTCGGGAGGTACATCAAGTAAAAGAAATGTTGGTGATC-3^′^ or 5^′^-GCGggatccGGTGGATCTGGTGGATCTGGTGGATCTGGTGGATCTGGTGGATCTGGTGGATCTGGCGGTAGTGGAGGCAGC-3^′^, and 5^′^-TGGCCGAgtcgacTCAGATGAATGCAAAAAGAAGAGAAATTAATG-3^′^. The fragments were then inserted into the *Bam*HI and *Sal*I sites of pYS0-sst, resulting in plasmids pYS-notag3-sst, pYS-notag20-sst, and pYS-notag38-sst.

Plasmids for producing membrane-displayed somatostatin with an alanine substitution (Table 2) were constructed as follows. A DNA fragment encoding the glucoamylase secretion signal and the modified somatostatin was amplified from pULD1 with primers 5^′^-CGGggtaccATGCAACTGTTCAATTTGCCATTG-3^′^ and either 5^′^-CGCggatccACCACAGGATGTGAAAGTCTTAGCGAAGAAATTCTTGCAGCCAGCGGCAGAAACGAGCAAAGAAAAG3^′^ or 5^′^-CGCggatccACCAGCGGATGTGAAAGTCTTCCAGAAGAAATTCTTGCAGCCAGCGGCAGAAACGAGCAAAGAAAAG-3^′^, and was inserted into the *Kpn*I and *Bam*HI sites in pYS21, generating the plasmids pYS21-sst (W8A) and pYS21-sst(C14A), respectively.

### Construction of yeast strains

All yeast transformation procedures were conducted with a Frozen-EZ Yeast Transformation-II kit (Zymo Research Corporation, Irvine, CA, USA). The yeast strains used and constructed in this study are listed in Table 1.

The WH-7 strain, which carried *SST2*, *FAR1*, and *STE2* disruptions, was obtained using the 2-step procedure described by Gueldener *et al.* (2002) as follows. The *STE2* of strain WH-3 (Hara et al. 2012a) was disrupted using the *loxP-URA3-loxP* cassette, which had been amplified from pUG72 (Gueldener et al. 2002) with the primers 5^′^-GTTACTTAAAAATGCACCGTTAAGAACCATATCCAAGAATCAAAAcagctgaagcttcgtacgc-3^′^ and 5^′^-ATACCGAAGGTCACGAAATTACTTTTTCAAAGCCGTAAATTTTGAgcataggccactagtggatctg-3^′^. Next, Ura- derivatives of the transformants were selected on media containing 5-fluoroorotic acid (Boeke et al. 1987). *STE2* disruption at the targeted locus and removal of *URA3* were verified by PCR using appropriate primers. *GPA1* disruption was conducted after introduction of p413-GPA1(Gi2) into the WH-7 strain because the absence of the Gα protein causes constitutive activity due to the existence of free Gβγ subunits (Guo et al. 2003). The *GPA1* of WH-7 with p413-GPA1(Gi2) was disrupted using the *KanMX4* cassette that had been amplified from the genomic DNA of strain BY4741 *gpa1Δ* (EUROSCARF, Frankfurt, Germany) with the primers 5^′^-ACAAGATCATAGGTGGATAAAGCAAGCCG-3^′^ and 5^′^-GCCTAGTAGATCTTGATTCTTTGTCACCTC-3^′^, to create strain WH-9. The WH-9 strain was transformed with p412-SSTR2 and a signal detection plasmid (pYEX-fet (Hara et al. 2012a)), resulting in the strain WSTH-9. The WSTH-9 strain was transformed with one of the plasmids for producing membrane-displayed somatostatin.

### Cultivation conditions

To prepare starter cultures, a series of WSTH-9 strains with one of the membrane-displayed ligand plasmids was grown in 10 mL of SC-HALW medium (pH 7.2) at 30°C overnight. The cultures used for assays were initiated by inoculating the starter culture into 10 mL SC-HALW medium to give an initial optical density (OD) of 0.05 at 600 nm using a VMax Kinetic ELISA Microplate Reader (Molecular Devices, Sunnyvale, CA, USA). These cultures were grown at 30°C on a rotary shaker (250 rpm) until the OD_600_ reached 0.5**–**0.6 corresponding to the late log phase.

For signal activation by exogenously added somatostatin, WSTH-9 strains (without the membrane-displayed ligand plasmid) were cocultured with serial dilutions of somatostatin, which was added at the start of the main culture. These cultures were also grown at 30°C on a rotary shaker (250 rpm) until the OD_600_ reached 0.5**–**0.6 corresponding to the late log phase.

### Plate assay

Two-hundred microliters of the main cultures with OD_600_ ranging over 0.5–0.6 were transferred to a 96-well plate (353072; BD Falcon, BD Biosciences, San Diego, CA, USA), and the fluorescence emitted was measured with a Fluoroskan Ascent Fluorometer (Labsystems OY, Helsinki, Finland). A filter pair with an excitation wavelength at 485 nm and emission wavelength at 527 nm was used to detect the fluorescence of enhanced green fluorescent protein (EGFP) produced in the yeast cells. The OD_600_ of each well of the sample plate was measured simultaneously. In order to normalize the number of cells in each well, the fluorescence value was divided by the OD_600_ reading. The fluorescence of the negative control (pYS0-nega strain, see subsection on **Expression vectors**) was subtracted from those for all of the other samples.

### Western blot analysis

A cell membrane fraction was prepared according to the previously described method (Frieman and Cormack 2004; Hara et al. 2012a). Membrane proteins were analyzed by electrophoresis on 15% (w/v) SDS polyacrylamide gels and electrotransferred onto a nitrocellulose membrane (Bio-Rad, Richmond, CA, USA). The membrane was pretreated with 5% (w/v) skim milk powder in PBS (pH 7.4), overnight, for preventing the nonspecific binding of antibody. The membrane was incubated for 1 h at room temperature with mouse anti-FLAG M2-Peroxidase antibody (Sigma-Aldrich, St Louis, MO, USA) at a concentration of 1:10000 in 5% skim milk/PBS. The blots were washed 5 times in 2% (v/v) Tween/PBS for 5 min, and antibody binding was detected using ECL-Plus (GE Healthcare, Buckinghamshire, UK).

## Results

**Figure 1:**
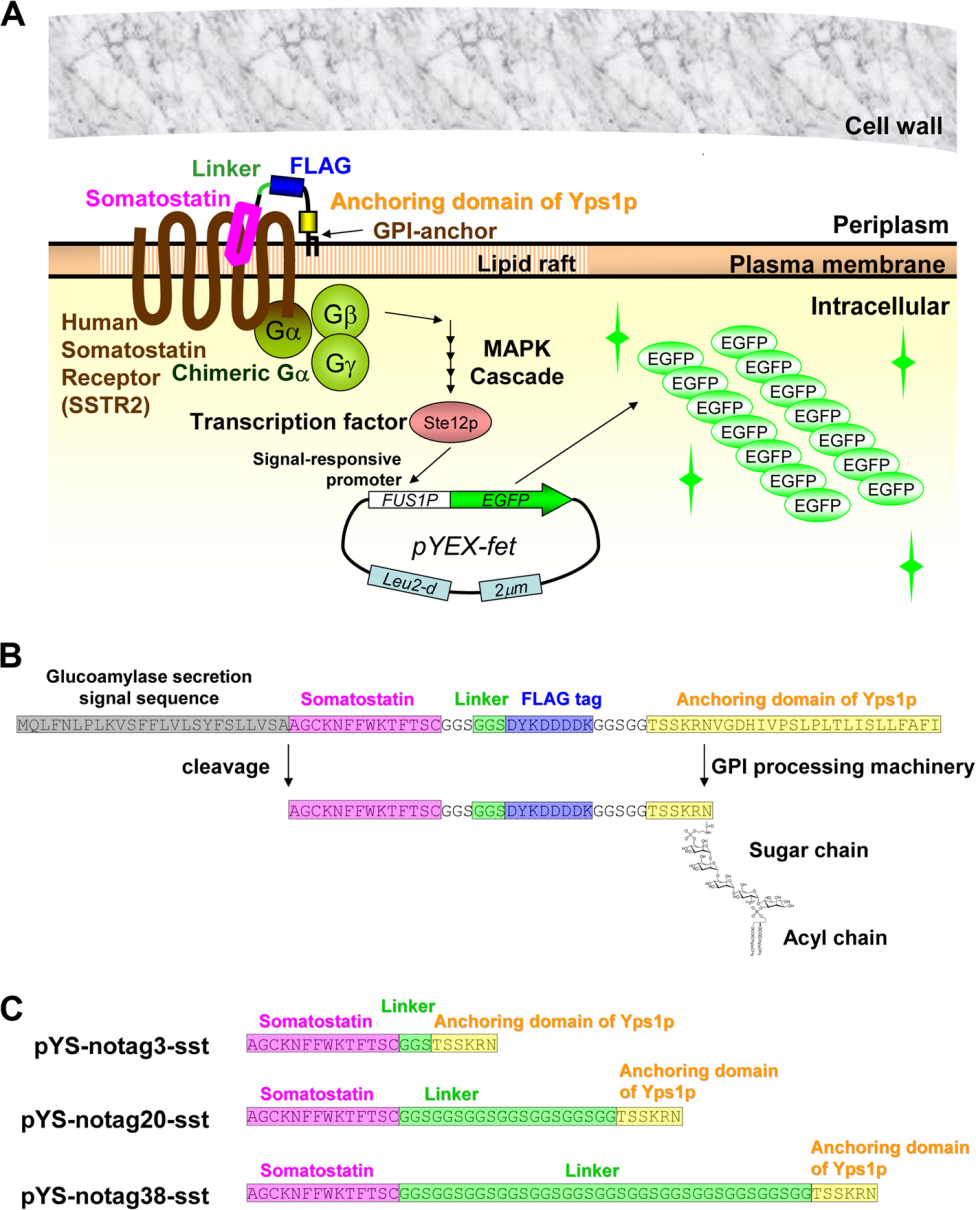
**Schematic representation of signal activation of human somatostatin receptor subtype-2 by membrane-displayed somatostatin. A**. Overview of this study. Membrane-displayed somatostatin (cyclic peptide) activates human somatostatin receptor subtype-2, which is heterologously produced in yeast, and leads to the activation of chimeric Gα proteins, the mitogen-activated protein kinase cascade, and transcription factor Ste12p. Phosphorylated Ste12p induces the overexpression of enhanced green fluorescent protein by binding to a pheromone response element in the *FUS1* promoter of pYEX-fet (signal detection plasmid) (Hara et al. 2012a). This expression allows monitoring and quantification of signal transduction by fluorescence. **B**. Functional domains encoded by the membrane-displayed somatostatin plasmid. After processing by the secretory pathway, the signal sequence and glycosylphosphatidylinositol (GPI) targeting sequence are cleaved, and the somatostatin sequence, with a free N-terminus is displayed on the plasma membrane by GPI covalently linked to the C-terminus. **C**. Amino-acid sequence of membrane-displayed somatostatin without FLAG tag.

**Figure 2:**
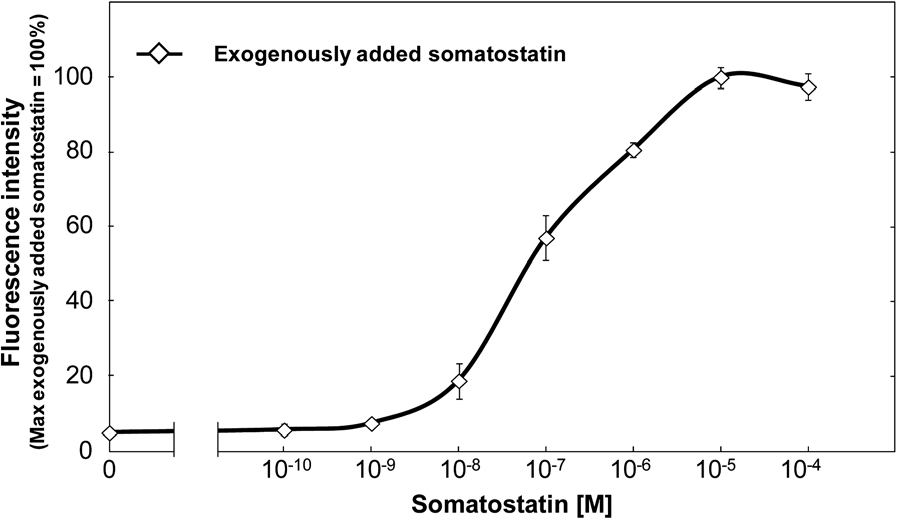
**Activation of human somatostatin receptor subtype-2 produced in yeast by exogenously added somatostatin. **Human somatostatin receptor subtype-2 was produced in yeast and stimulated by exogenously added somatostatin at various concentrations. The fluorescence intensity derived from receptor activation induced by enhanced green fluorescent protein was measured by using a 96-well fluorescent plate reader. Each data point represents the data (mean ± standard error of the mean) of three independent experiments.

An overview of this study is illustrated in Figure 1. To optimize the signal output, gene deletions were performed on the negative regulator of Gpa1 activity (*sst2Δ*) and a cyclin-dependent kinase inhibitor (*far1Δ*). For the production of human SSTR2 and chimeric Gα subunit, gene deletions were performed on yeast endogenous GPCR (*ste2Δ*) and G-protein α-subunit (*gpa1Δ*) accompanied by the introduction of human SSTR2 expression plasmid and chimeric Gα expression plasmid (Brown et al. 2000; Hara et al. 2012b). The chimeric Gα subunit employed in this study was the “transplant” type, in which the C-terminal five amino acids of Gpa1p (KIGII) were exchanged with the mammalian Gi sequence (DCGLF). Signal activation was detected by fluorescence from pYEX-fet (Hara et al. 2012a), which is the high copy number reporter plasmid that contains *EGFP* and is controlled by the signal responsive *FUS1* promoter. When the constructed WSTH-9 strain (Table 1) was exposed to exogenously added somatostatin, signal activation was observed in a dose-dependent manner (Figure 2), indicating that the chimeric Gα and human SSTR2 that were heterologously produced in the yeast were functional.

**Figure 3:**
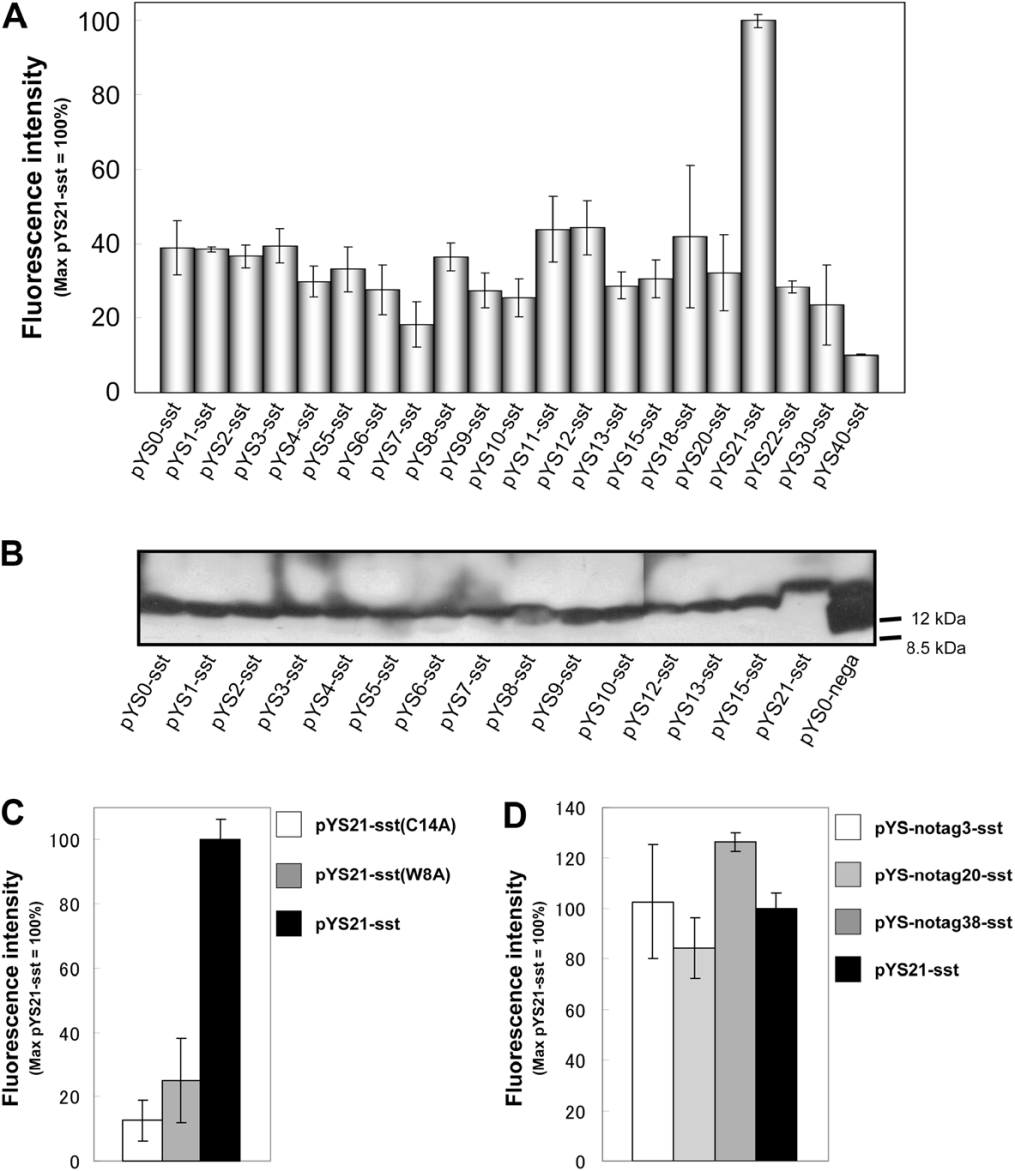
**Activation of human somatostatin receptor subtype-2 by membrane-displayed somatostatin. A**. Fluorescence intensity of yeast strains transformed with membrane-displayed somatostatin plasmids. A series of yeast strains with one of the membrane-displayed ligand plasmids was assayed in the late-log phase using a 96-well fluorescent plate reader. Yeast cells harboring pYS21-sst emitted strong fluorescence. Each data point represents data (mean ± standard error of the mean) of three independent experiments. **B**. Western blot analysis of a series of membrane-displayed somatostatins. Production of membrane-displayed somatostatin on yeast plasma membranes was confirmed by western blot analysis using anti-FLAG tag antibody. The positions of 8.5 and 12 kDa of size markers are indicated by black bars. **C**. Fluorescence intensity of yeast strains transformed with plasmids for membrane-displayed somatostatin with alanine substitution. Each data point represents data (mean ± standard error of the mean) of three independent experiments. **D**. Linker dependency of membrane-displayed somatostatin without FLAG tag upon receptor activation. Fluorescence intensity of yeast strains transformed with plasmids for membrane-displayed somatostatin without FLAG tag. Each data point represents data (mean ± standard error of the mean) of three independent experiments.

We introduced plasmids for the membrane display of somatostatin with different linker lengths into the WSTH-9 strain. The fluorescence intensity and OD_600_ of the cell culture were measured at the late log phase, and the fluorescence intensities of each strain divided by the OD_600_ were compared (Figure 3A). Among the broad spectrum of activities in a series of yeast strains that possessed one of the membrane-displayed somatostatin plasmids, a strain with pYS21-sst exhibited the strongest signal (Figure 3A). In addition, the production of membrane-displayed somatostatin with various linker lengths on the yeast plasma membrane was confirmed by western blot analysis of the membrane fraction using anti-FLAG tag antibody (Figure 3B). These results indicate that the membrane-displayed somatostatin directly activated the human SSTR2.

To confirm whether the signal activation triggered by the membrane-displayed somatostatin was due to the displayed somatostatin, alanine substitution into somatostatin was employed (Figure 3C). Trp^8^ is critically important for agonistic activity and alanine substitution at Trp^8^ causes a significant loss in activity (Rosenthal et al. 1983; Vale et al. 1978). When Trp^8^ of the membrane-displayed somatostatin (pYS21-sst) was substituted by alanine, the activity was significantly decreased (Figure 3C). This indicates that the activation was triggered by the somatostatin that was displayed on plasma membrane. Somatostatin is a naturally occurring cyclic peptide hormone with a disulfide bond between Cys^3^ and Cys^14^ residues. It has been reported that the hormonal activity was lost in a linearized form of somatostatin (Vale et al. 1978). To confirm that the membrane-displayed somatostatin retained its cyclic structure, the alanine-substituted form of membrane-displayed somatostatin at Cys^14^ was also constructed. Figure 3C shows that the strain harboring pYS21-sst(C14A) significantly lost its activity, suggesting that the membrane-displayed somatostatin had a cyclic structure with a disulfide bond between Cys^3^ and Cys^14^ residues.

From the results of Figure 3A, the signal intensity of each strain depended on the linker length. One of the reasons for this linker length dependence could be the FLAG tag sequence (DYKDDDDK), which possesses a negatively charged sequence. It is possible that this region interacts with the relatively polar amino acids on the outer loop of GPCRs, which could give rise to linker dependency. Considering that the linker dependency observed in Figure 3A influences the robustness of this technology, membrane-displayed somatostatins without a FLAG tag were constructed to overcome this problem. The linker lengths between somatostatin and the membrane-anchoring domain of Yps1p were designed to have 3, 20, and 38 amino acids (pYS-notag3-sst, pYS-notag20-sst, and pYS-notag38-sst, respectively). Regardless of the linker lengths, the membrane-displayed somatostatins without FLAG tag had activity similar to pYS21-sst (Figure 3D). This indicates that the negatively charged nature or the secondary structure of the FLAG tag region could have resulted in the linker dependency shown in Figure 3A, and that removal of FLAG tag led to the reduction of linker dependency of this system.

## Discussion

We applied our technology for displaying peptide ligands on a plasma membrane to human SSTR2 that was heterologously produced in yeast cells. Functional activation of the pheromone response pathway by the membrane-displayed somatostatin was successfully achieved. In spite of the previous reports of the functional production of rat SSTR2 (Price et al. 1995) and human SSTR5 (Fukuda et al. 2011) in yeast, this is the first report that documents the functional production of human SSTR2 in yeast. While cell wall trapping of autocrine peptides strategy, in which peptide ligands were displayed on yeast cell wall, has recently been reported (Ishii et al. 2012), the percentage of activated cells by the system was lower than that by our system (Hara et al. 2012a). This suggests that plasma membrane is more suitable than cell wall in terms of peptide-ligand display. The higher accessibility of membrane-displayed ligands to GPCRs may be important for efficient activation. Here, we termed “PepDisplay” as a technology for displaying peptide ligands on a plasma membrane for activation of either endogenous or heterologous GPCRs produced in yeast cells.

In order to estimate the signal intensity triggered by the membrane-displayed somatostatin, the signal intensity of strain pYS-notag38-sst was compared with that of exogenously added somatostatin treatment (Figure 2). The signal intensity was found to be 86.6% of the maximum response of the exogenously added somatostatin treatment (data not shown). Considering that tethered peptide-induced signal activation ranged from 35% to 100% of the soluble hormone-induced maximum intensity in a mammalian experimental system (Fortin et al. 2009), the intensity obtained in this study is reasonable. Although the signal intensity can be improved by further scrutiny (e.g., length and amino-acid composition of the linker region, and expression level of the membrane-displayed ligand), the signal intensity obtained by pYS-notag38-sst was sufficient for detection and sorting by fluorescence-activated cell sorter (Hara et al. 2012a).

To the best of our knowledge, the functional display of cyclic peptides on the plasma membrane has not been previously reported although several research groups have reported on membrane-tethered ligands (Chang et al. 2009; Choi et al. 2009; Fortin et al. 2011; Fortin et al. 2009). Cyclic peptides comprise a promising type of scaffold for peptide therapeutics (Katoh et al. 2011), and the linker dependency of PepDisplay was solved by eliminating the FLAG tag; hence, the methodology presented here will provide a new platform for identifying novel peptide ligands for both liganded and orphan mammalian GPCRs.

## Abbreviations

GPCR: G protein-coupled receptor; GPI: Glycosylphosphatidylinositol; EGFP: Enhanced green fluorescent protein; OD: Optical density; SC: Synthetic complete; SSTRs: Somatostatin receptors; SSTR2: Somatostatin receptor subtype-2.

## Competing interests

The authors declare that they have no competing interests.
